# Supplementary material for: Cross-cultural adaptation and measurement properties of the French version of the Trinity Amputation and Prosthesis Experience Scales—Revised (TAPES-R)
Source: PLoS One. 2020 Feb 21;15(2):e0229084. doi: 10.1371/journal.pone.0229084 (PMC7034834; doi:10.1371/journal.pone.0229084)
Supplement: S1 Data — (PDF) [file pone.0229084.s001.pdf]

TAPES-R-F

Ce questionnaire a pour but de mesurer la façon dont vous vous êtes adapté(e) à votre amputation, l'influence de l'amputation sur vos activités quotidiennes ainsi que votre degré de satisfaction avec votre prothèse. Ce n'est pas un test, il n'y a pas de réponse juste ou fausse. Merci de répondre aussi précisément que vous le pouvez à chacune des questions, en cochant la case qui correspond le mieux à ce que vous pensez :

**Adaptation psycho-sociale**	Pas du tout d'accord	Pas d'accord	D'accord	Totalement d'accord
1. Je me suis adapté(e) à avoir une prothèse	☐	☐	☐	☐
2. Avec le temps qui passe, j'accepte mieux ma prothèse	☐	☐	☐	☐
3. J'ai le sentiment d'avoir réussi à gérer ce traumatisme dans ma vie	☐	☐	☐	☐
4. Même si j'ai une prothèse, ma vie est bien remplie	☐	☐	☐	☐
5. Je me suis habitué(e) à porter une prothèse	☐	☐	☐	☐
6. Ça m'est égal si quelqu'un regarde ma prothèse	☐	☐	☐	☐
7. Je parle facilement de ma prothèse	☐	☐	☐	☐
8. Ça ne me dérange pas si quelqu'un me pose des questions sur ma prothèse	☐	☐	☐	☐
9. Je parle facilement avec les autres de mon membre perdu	☐	☐	☐	☐
10. Ça m'est égal si quelqu'un remarque que je boite	☐	☐	☐	☐
11. Une prothèse limite mes capacités à faire mon travail	☐	☐	☐	☐
12. Avoir une prothèse me rend plus dépendant des autres que ce que je voudrais	☐	☐	☐	☐
13. Avoir une prothèse limite le genre de travail que je peux faire	☐	☐	☐	☐
14. Etre amputé(e) signifie que je ne peux pas faire ce que je veux faire	☐	☐	☐	☐
15. Avoir une prothèse limite la quantité de travail que je peux faire	☐	☐	☐	☐

**Limitation des activités**	Oui, très limité	Un peu limité	Non, pas du tout limité
a. Des activités énergiques telles que courir, soulever des objets lourds, pratiquer des sports exigeants physiquement	☐	☐	☐
b. Monter plusieurs étages d'escaliers	☐	☐	☐
c. Courir pour attraper un bus	☐	☐	☐
d. Sports et loisirs	☐	☐	☐
e. Monter un étage d'escaliers	☐	☐	☐
f. Marcher plus d' 1 km	☐	☐	☐
g. Marcher 500 m	☐	☐	☐
h. Marcher 100 m	☐	☐	☐
i. Pratiquer mes passe-temps	☐	☐	☐
j. Travailler	☐	☐	☐

**Satisfaction avec la prothèse**	Insatisfait	Satisfait	Très satisfait
i. Couleur	☐	☐	☐
ii. Forme	☐	☐	☐
iii. Apparence	☐	☐	☐
iv. Poids	☐	☐	☐
v. Utilité	☐	☐	☐
vi. Fiabilité	☐	☐	☐
vii. Ajustement	☐	☐	☐
viii. Confort	☐	☐	☐

Merci de mettre une croix dans la case qui décrit le mieux votre degré de satisfaction avec votre prothèse

	0	1	2	3	4	5	6	7	8	9	10	
Pas du tout satisfait	☐	☐	☐	☐	☐	☐	☐	☐	☐	☐	☐	Très satisfait
