# Supplementary material for: Cross-cultural adaptation and measurement properties of the French version of the Trinity Amputation and Prosthesis Experience Scales—Revised (TAPES-R)
Source: PLoS One. 2020 Feb 21;15(2):e0229084. doi: 10.1371/journal.pone.0229084 (PMC7034834; doi:10.1371/journal.pone.0229084)
Supplement: S2 Data — (PDF) [file pone.0229084.s002.pdf]

Code ☐☐☐☐☐☐ ☐☐ Date ☐☐ / ☐☐ / ☐☐☐☐

TAPES-R

This is a questionnaire designed to investigate different aspects of having a prosthesis. There are no right or wrong answers. Please answer every item as honestly as you can. For each question, please tick clearly inside one box using a black or blue pen.

	**Strongly disagree**	**Disagree**	**Agree**	**Strongly agree**
1. I have adjusted to having a prosthesis	☐	☐	☐	☐
2. As time goes by, I accept my prosthesis more	☐	☐	☐	☐
3. I feel that I have dealt successfully with this trauma in my life	☐	☐	☐	☐
4. Although I have a prosthesis, my life is full	☐	☐	☐	☐
5. I have gotten used to wearing a prosthesis	☐	☐	☐	☐
6. I don’t care if somebody looks at my prosthesis	☐	☐	☐	☐
7. I find it easy to talk about my prosthesis	☐	☐	☐	☐
8. I don’t mind people asking about my prosthesis	☐	☐	☐	☐
9. I find it easy to talk about my limb loss in conversation	☐	☐	☐	☐
10. I don’t care if somebody notices that I am limping	☐	☐	☐	☐
11. A prosthesis interferes with the ability to do my work	☐	☐	☐	☐
12. Having a prosthesis makes me more dependent on others than I would like to be	☐	☐	☐	☐
13. Having a prosthesis limits the kind of work that I can do	☐	☐	☐	☐
14. Being an amputee means that I can’t do what I want to do	☐	☐	☐	☐
15. Having a prosthesis limits the amount of work that I can do	☐	☐	☐	☐

Limitation	Yes, limited a lot	Limited a little	No, not limited at all
a. vigorous activities, such as running, lifting heavy objects, participating in strenuous sports	☐	☐	☐
b. climbing several flights of stairs	☐	☐	☐
c. running for a bus	☐	☐	☐
d. sport and recreation	☐	☐	☐
e. climbing one flight of stairs	☐	☐	☐
f. walking more than a mile	☐	☐	☐
g. walking half a mile	☐	☐	☐
h. walking 100 metres	☐	☐	☐
i. working on hobbies	☐	☐	☐
j. going to work	☐	☐	☐

Satisfaction	Not satisfied	Satisfied	Very satisfied
i. Colour	☐	☐	☐
ii. Shape	☐	☐	☐
iii. Appearance	☐	☐	☐
iv. Weight	☐	☐	☐
v. Usefulness	☐	☐	☐
vi. Reliability	☐	☐	☐
vii. Fit	☐	☐	☐
viii. Comfort	☐	☐	☐

Please tick the box (0-10) that best describes how satisfied you are with your prosthesis?

	**0**	**1**	**2**	**3**	**4**	**5**	**6**	**7**	**8**	**9**	**10**	
Not at all satisfied	☐	☐	☐	☐	☐	☐	☐	☐	☐	☐	☐	Very satisfied
