# Supplementary material for: Cross-cultural adaptation and measurement properties of the French version of the Trinity Amputation and Prosthesis Experience Scales—Revised (TAPES-R)
Source: PLoS One. 2020 Feb 21;15(2):e0229084. doi: 10.1371/journal.pone.0229084 (PMC7034834; doi:10.1371/journal.pone.0229084)
Supplement: Attachment — Submitted filename: Comments_Cross-cultural adaptation and measurement properties of the French version of the Trinity Amputation and Prosthesis Experience Scales -Revised (TAPES-R).docx [file pone.0229084.s004.docx]

Comments_Cross-cultural adaptation and measurement properties of the French version of the Trinity Amputation and Prosthesis Experience Scales -Revised (TAPES-R)

Dear Authors,

I am grateful for the efforts to validate TAPES-R in the French language. The design of the research, collection and analysis of data, and presentation of the findings are straightforward and scientifically rigor. The steps of cultural adaptation and validation process are thoroughly followed by the authors. The reviewer acknowledges the practical relevance and research advancements of this research, which will allow many practitioners to measure the outcomes of their support and understand the overall wellbeing of the people affected by amputation.

The reviewer recommends publishing this research, after reviewing and adjusting a few methodological and practical comments outlined below.

1. *To date, there is no validated version of the TAPES-R in another language, as all previous translations in German, Persian and Turkish have been made from the first version of the TAPES*. (Page 11)

- Kind request to review a recent study of Massarweh and Sobuh (2019) for TAPES-R Arabic version and adjust accordingly.

2. *Conversely, the inability to read or understand French, being a resident in a health or retirement home was the only exclusion criteria*. (page 11)

- The reasons for excluding the participants from retirement homes should have been mentioned. Mostly the people living in retirement homes are single and without family/social support. The studies have explored a higher level of anxiety and depression in people with amputation who are alone (Parkes, 1976; Hawamdeh, Othman, & Ibrahim, 2008).

3. In any validation studies of a psychological tool, the sample size remains crucial. The authors could not explain the nature of the sample size, it seems, a convenience sample was used. The sample size determination techniques would have been better explained either in the main section or in limitations (Etikan, Musa, & Alkassim, 2016). For the factor analysis, a minimum of 6-10 samples per item of measure is required to draw complete factor loads and measurement fits of the data. As confirmatory factor analysis was not carried out, the EFA and Rasch analysis were presented. The results received from Rasch analysis, for less than 100 samples, have inadequate and sometimes opposite conclusions (Chen et al., 2014). Therefore, the reviewer recommends the authors to address the sample methods, sample size, and interrelated issues. Kaiser-Meyer-Olkin (KMO) sampling adequacy test results would have been best to present if possible.
4. The researchers are also requested to state/describe the normality test outcomes of the data, because the result section does not include it. However, the data analysis section of methodology outlines it.
5. *All the five items should be associated with general adjustment. Construct validity is considered acceptable if at least 75% of the results are in correspondence with our hypotheses, which would here correspond to 34/46 (Page 17)*

- The presentation of construct validity outcomes seems very vague, cannot be well understandable. The sentence, *‘Construct validity is considered acceptable if at least 75% of the results are in correspondence with our hypotheses, which would here correspond to 34/46’,* needs further elaboration.

6. It would be better to provide factor loadings of all items of each measure, so that the readers will understand better (Page 21 under the factor analysis).
7. *The present result would rather suggest to further reduce the rating to three categories for the general adjustment subscale, but leave it as is all other subscales. Nevertheless, since the three subscales on psychosocial adjustment share the same section of the questionnaire, it seems wiser to leave all items as is.*

- I would have deleted the highlighted text in Yellow.

References:

Massarweh, R., & Sobuh, M. M. (2019). The Arabic Version of Trinity Amputation and Prosthetic Experience Scale-Revised (TAPES-R) for Lower Limb Amputees: Reliability and Validity. Disability, CBR & Inclusive Development, 30(1), 44-56. doi: 10.5463/dcid.v30i1.718

Gallagher, P., & MacLachlan, M. (2004). The Trinity Amputation and Prosthesis Experience Scales and quality of life in people with lower-limb amputation. Archives of Physical Medicine and Rehabilitation, 85(5), 730-736. doi: 10.1016/j.apmr.2003.07.009

Arafat, S. (2016). Cross cultural adaptation & psychometric validation of instruments: Step-wise description. International Journal of Psychiatry, 1(1), 1-4. doi: 10.33140/IJP/01/01/00001

Parkes, C. M. (1976). The psychological reaction to loss of a limb: the first year after amputation. In Modern perspectives in the psychiatric aspects of surgery (pp. 515-532). Palgrave Macmillan UK.

Hawamdeh, Z. M., Othman, Y. S., & Ibrahim, A. I. (2008). Assessment of anxiety and depression after lower limb amputation in Jordanian patients. Neuropsychiatric disease and treatment, 4(3), 627-633. doi: 10.2147/NDT.S2541

Etikan, I., Musa, S. A., & Alkassim, R. S. (2016). Comparison of convenience sampling and purposive sampling. American journal of theoretical and applied statistics, 5(1), 1-4. doi: 10.11648/j.ajtas.20160501.11

Chen, W. H., Lenderking, W., Jin, Y., Wyrwich, K. W., Gelhorn, H., & Revicki, D. A. (2014). Is Rasch model analysis applicable in small sample size pilot studies for assessing item characteristics? An example using PROMIS pain behavior item bank data. Quality of life research, 23(2), 485-493. doi: 10.1007/s11136-013-0487-5
