# Supplementary material for: Cross-cultural adaptation and measurement properties of the French version of the Trinity Amputation and Prosthesis Experience Scales—Revised (TAPES-R)
Source: PLoS One. 2020 Feb 21;15(2):e0229084. doi: 10.1371/journal.pone.0229084 (PMC7034834; doi:10.1371/journal.pone.0229084)
Supplement: Attachment — Submitted filename: Response to Reviewers.docx [file pone.0229084.s005.docx]

| Reviewer Remarks | Authors’ Responses | Text Changes |
| --- | --- | --- |

| Reviewer 1 |  | Track Changes |
| --- | --- | --- |
| 1. To date, there is no validated version of the TAPES-R in another language, as all previous translations in German, Persian and Turkish have been made from the first version of the TAPES. (Page 11)  - Kind request to review a recent study of Massarweh and Sobuh (2019) for TAPES-R Arabic version and adjust accordingly. | Thank you for your remark. We were not aware of this very recent article when we wrote the manuscript. It has been added to the bibliography and discussed in the manuscript.  We mentioned the Arabic version (Massarweh et al., 2019) in the Introduction, compared our results with those of this translation (dimensionality, internal consistency) and made some comments in the Discussion. | Introduction (l.33-4)  Methods (l. 239)  Factor analysis (l.278-9)  Cronbach alpha (l. 308-10)  Discussion (l. 413-15, and l. 418-19) |
| 2. Conversely, the inability to read or understand French, being a resident in a health or retirement home was the only exclusion criteria. (page 11)  - The reasons for excluding the participants from retirement homes should have been mentioned. Mostly the people living in retirement homes are single and without family/social support. The studies have explored a higher level of anxiety and depression in people with amputation who are alone (Parkes, 1976; Hawamdeh, Othman, & Ibrahim, 2008). | We agree with your remark, but the reason why people living in retirement home were excluded from this study is simple. This TAPES-R validation study will be part of an ongoing larger study on the quality of life, participation and integration of amputees returning to live at home after lower limb amputation in Switzerland. Living in a retirement home was therefore the reason for exclusion. According to your remark, we added precisions on people living in retirement home in the discussion. | Discussion (l.505-8) |
| 3. a) In any validation studies of a psychological tool, the sample size remains crucial. The authors could not explain the nature of the sample size, it seems, a convenience sample was used.   b) The sample size determination techniques would have been better explained either in the main section or in limitations (Etikan, Musa, & Alkassim, 2016).  c) For the factor analysis, a minimum of 6-10 samples per item of measure is required to draw complete factor loads and measurement fits of the data.  d) As confirmatory factor analysis was not carried out, the EFA and Rasch analysis were presented.  e) The results received from Rasch analysis, for less than 100 samples, have inadequate and sometimes opposite conclusions (Chen et al., 2014). Therefore, the reviewer recommends the authors to address the sample methods, sample size, and interrelated issues.  f) Kaiser-Meyer-Olkin (KMO) sampling adequacy test results would have been best to present if possible. | a) Convenience sampling was used to include the patients, as already mentioned in the Results section (l.242).  b) We add the sampling determination as a limitation, since we could not ensure that our convenient sample is representative of the whole population of amputees, which limit the ability to draw inferences about the population (Etikan et al., 2016).  c) Factor analysis was performed on each scale of the TAPES-R-F separately, which means that the largest number of items was 15 (psychosocial adjustment scale). Our sample size of n=99 satisfies the requirement of 6 subjects per item. We also add KMO test (see hereafter) that confirmed the adequacy of this sample size.  d) Both EFA and CFA were performed. This has been clarified in the text.  e) We agree with the reviewer that our sample size is a bit small (although n=99 is not so far from 100). Nevertheless, Chen et al. (2014) observed inadequate conclusions especially in smaller samples (30, 50). In the Discussion, they mentioned that “The simulations demonstrated that there were more stable estimates observed in samples that were 100 or greater.” Moreover, their recommendation is “that researchers not to make any definitive decisions about the items, such as deleting items or collapsing response categories, based on the results from a small sample.” We followed this recommendation when leaving the rating categories for the general adjustment subscale as is, despite the results of the Rasch analysis. This point was added in the Results section.  f) Thank you for this suggestion. KMO test was added before the FA results. | a) No text change  b) Discussion (L 497-500)  c) No text change  d) Results (l. 272 and l. 279)  e) Results (l. 299-300)  f) Results (l. 265-271) |
| 4. The researchers are also requested to state/describe the normality test outcomes of the data, because the result section does not include it. However, the data analysis section of methodology outlines it. | As mentioned in the Methods section, no normality test was performed, but a visual inspection of the distribution. We added in the Results section that the normality assumption was satisfied.  We did not conduct any normality test. The reason is methodological: with a small sample size, the test will not be powerful enough to reject the null hypothesis of normality whatever the distribution is, wrongly suggesting that the distribution can be considered normal. In the opposite case of a large sample size, the test will almost surely reject the null hypothesis of normality, even if the distribution is very close to normal. | Methods (l.177)  Results (l.314-15) |
| 5. All the five items should be associated with general adjustment. Construct validity is considered acceptable if at least 75% of the results are in correspondence with our hypotheses, which would here correspond to 34/46 (Page 17)  - The presentation of construct validity outcomes seems very vague, cannot be well understandable. The sentence, ‘Construct validity is considered acceptable if at least 75% of the results are in correspondence with our hypotheses, which would here correspond to 34/46’, needs further elaboration. | According to Terwee (for details see: Terwee et al., 2007. Quality criteria for measurement properties of health status questionnaires. J Clin Epidemiol) construct validity is considered as adequate if at least 75% of a priori hypotheses are then confirmed. In the methods, we detailed all the associations we expected to find between the TAPES-R and the other questionnaires used to measure construct validity. The sum of these possible associations, considered as our a priori hypotheses, represented 46 hypotheses. The criterion of at least 75% thus corresponds to at least 35/46 confirmed hypotheses in the results. | Methods  (l. 199-200)  Results (l. 357-8) |
| 6. It would be better to provide factor loadings of all items of each measure, so that the readers will understand better (Page 21 under the factor analysis). | A Table with the factor loadings corresponding to the psychosocial adjustment scale has been added in the Results section. Since the other two scales (activity restriction and satisfaction with the prosthesis) consist of one single factor, we judged useless to provide the corresponding factor loadings. | Results (l. 280-6) |
| 7. The present result would rather suggest to further reduce the rating to three categories for the general adjustment subscale, but leave it as is all other subscales. Nevertheless, since the three subscales on psychosocial adjustment share the same section of the questionnaire, it seems wiser to leave all items as is.  - I would have deleted the highlighted text in Yellow. | Done. The highlighted text was deleted | Results (l.296-8) |
